# Branched PCL-Based Thermogelling Copolymers: Controlling Polymer Architecture to Tune Drug Release Profiles

**DOI:** 10.3389/fbioe.2022.864372

**Published:** 2022-03-30

**Authors:** Qianyu Lin, Valerie Ow, Yi Jian Boo, Vincent T. A. Teo, Joey H. M. Wong, Rebekah P. T. Tan, Kun Xue, Jason Y. C. Lim, Xian Jun Loh

**Affiliations:** ^1^ NUS Graduate School for Integrative Sciences and Engineering, National University of Singapore (NUS), Singapore, Singapore; ^2^ Institute of Materials Research and Engineering (IMRE), Agency for Science, Technology and Research (A*STAR), Singapore, Singapore; ^3^ School of Materials Science and Engineering, Nanyang Technological University, Singapore, Singapore; ^4^ Department of Materials Science and Engineering, National University of Singapore (NUS), Singapore, Singapore

**Keywords:** polyurethane, amphiphilic, non-linear architecture, gel depot, sustained localized drug release, bovine serum albumin, model hydrophobic drug

## Abstract

Temperature-responsive hydrogels, or thermogels, are a unique class of biomaterials that show facile and spontaneous transition from solution to gel when warmed. Their high biocompatibility, and ease of formulation with both small molecule drugs and biologics have made these materials prime candidates as injectable gel depots for sustained local drug delivery. At present, controlling the kinetics and profile of drug release from thermogels is achieved mainly by varying the ratio of hydrophobic: hydrophilic composition and the polymer molecular weight. Herein, we introduce polymer branching as a hitherto-overlooked polymer design parameter that exhibits profound influences on the rate and profile of drug release. Through a family of amphiphilic thermogelling polymers with systematic variations in degree of branching, we demonstrate that more highly-branched polymers are able to pack less efficiently with each other during thermogel formation, with implications on their physical properties and stability towards gel erosion. This in turn resulted in faster rates of release for both encapsulated small molecule hydrophobic drug and protein. Our results demonstrate the possibility of exploiting polymer branching as a hitherto-overlooked design parameter for tailoring the kinetics and profile of drug release in injectable thermogel depots.

## Introduction

Thermogels are a unique class of supramolecular hydrogels that undergo reversible sol-gel phase transition when warmed (Moon et al., 2012; Constantinou and Georgiou, 2016; Cook et al., 2021; Lin et al., 2021). The thermogel is in the sol phase at low temperatures and transits to gel phase when heated above its gelation temperature. This gelation process is driven by the spontaneous micellization of the thermogelling amphiphilic copolymers, which further self-assembles via hydrophobic association and hydrogen bonding into a supramolecular matrix. Due to its facile reversible temperature controlled gelation, injectability, and high biocompatibility, thermogels have been intensely developed for biomedical applications such as sustained drug release platforms (Loh, 2018a), wound healing (Yun et al., 2012; Xu et al., 2019; Luo et al., 2020), and three dimensional scaffolds for cell culture (Hong et al., 2017; Patel et al., 2018; Constantinou and Georgiou, 2021). More recently, our group has successfully demonstrated thermogels as vitreous endotamponades in rabbit models (Liu et al., 2019; Xue et al., 2020a; Xue et al., 2020b).

Most commonly, thermogelling amphiphilic copolymers have di-block (Celik et al., 2018; Cui et al., 2018), tri-block (Dou et al., 2016; Smith et al., 2021), or multi-block (Loh et al., 2008; Loh et al., 2009) linear architectures. These copolymers often contain hydrophilic poly (ethylene glycol) (PEG) and hydrophobic segments such as poly (propylene glycol) (PPG), poly (ε-caprolactone) (PCL), poly (lactic-co-glycolic acid) (PLGA), poly (*N*-isopropylacrylamide) (PNiPAAm), and 2-(dimethylamino) ethyl methacrylate (DMAEMA). It is the dehydration of the hydrophobic segments above their lower critical solution temperature (LCST) that drives the micellization of these amphiphilic thermogelling copolymers and subsequently the self-assembly of the supramolecular hydrogel matrix by the aggregation of the micelles (Liow et al., 2016a).

Thermogels are especially useful for localised drug release applications as they can be injected as a chilled solution to the site of therapy, where rapid gelation then occurs to form a localised drug depot, triggered by body heat without additional chemical crosslinking (Cao et al., 2019; Chan et al., 2019; Yu et al., 2022). Indeed, the sustained and localised delivery of hydrophobic drugs for chemotherapy is particularly in demand (Chao et al., 2020). Other than enhancing the solubility of hydrophobic drugs in their micelle cores, thermogels can reduce side effects from the systematic circulation of drug by prolonging drug release at the localised site at therapeutic concentrations (Jiang et al., 2019). In addition to hydrophobic drugs, protein based therapies have also gained traction in the recent years (Delplace et al., 2019; Hogan and Mikos, 2020). In this regard, thermogels are also prime candidates as protein delivery platforms (Koshy et al., 2018; Cheng et al., 2020) as they are able to encapsulate proteins, protect them from denaturation, and localise their delivery for a prolonged duration (Ko et al., 2015; Dutta et al., 2020).

The kinetics and release profiles of drugs and proteins from thermogels are strongly influenced by copolymer composition (Shi et al., 2019; Xue et al., 2019) and molecular weight (Loh et al., 2012; Shah et al., 2018). In general, higher hydrophobic thermogel copolymer composition leads to increased encapsulation of hydrophobic drugs in the micelle cores, thus prolonging drug release by slowing thermogel erosion (Zheng et al., 2017; Jiang et al., 2019). In the case of hydrophilic proteins, they are mostly entrapped in the supramolecular matrix pores of the thermogel and their rate of release is dominated by diffusion (Delplace et al., 2019). As higher hydrophobic content in thermogelling copolymer may enlarge the pore sizes, the rate of protein release may thus be accelerated due to enhanced diffusion (Xue et al., 2019). On the other hand, higher molecular weight of thermogelling copolymers leads to both increased hydrophobicity and smaller pore sizes and would therefore prolong the release of both hydrophobic drugs and hydrophilic proteins (Loh et al., 2012; Xue et al., 2019).

In addition to these factors, our group has recently demonstrated that polymer branching is another important parameter in thermogel copolymer design that has profound impact on bulk thermogel properties, including storage moduli, gelation temperatures and rate of gel erosion (Lin et al., 2022). Similarly, previous studies by Li et al. (2012) and Teodorescu et al. (2010) on branched thermogelling copolymers also suggest that the number of branches and average length of the branches are both key factors that affect the characteristic of the resultant thermogels. However, the influence of polymer architecture (i.e., linear vs. branched) on the sustained release of hydrophobic small molecule and protein drugs has yet to be systematically explored. To address this deficiency, we synthesised a series of transparent and biodegradable PCL-based thermogels formulated from branched copolymers, and investigated how different degrees of branching influenced the release profiles of a model hydrophobic drug (fluorescein free acid, FA) and model hydrophilic protein (bovine serum albumin, BSA). We demonstrate that more extensive polymer branching resulted in accelerated release of encapsulated cargo by affecting thermogel microstructures and erosion rates, attributed to increased steric hindrance experienced by more branched copolymers during self-assembly. Our findings demonstrate that branching can be a novel polymer design consideration to control the release profiles of drugs and proteins from thermogels.

## Results and Discussion

### Synthesis and Characterisation of Branched Copolymers and Thermogels

**FIGURE 1:**
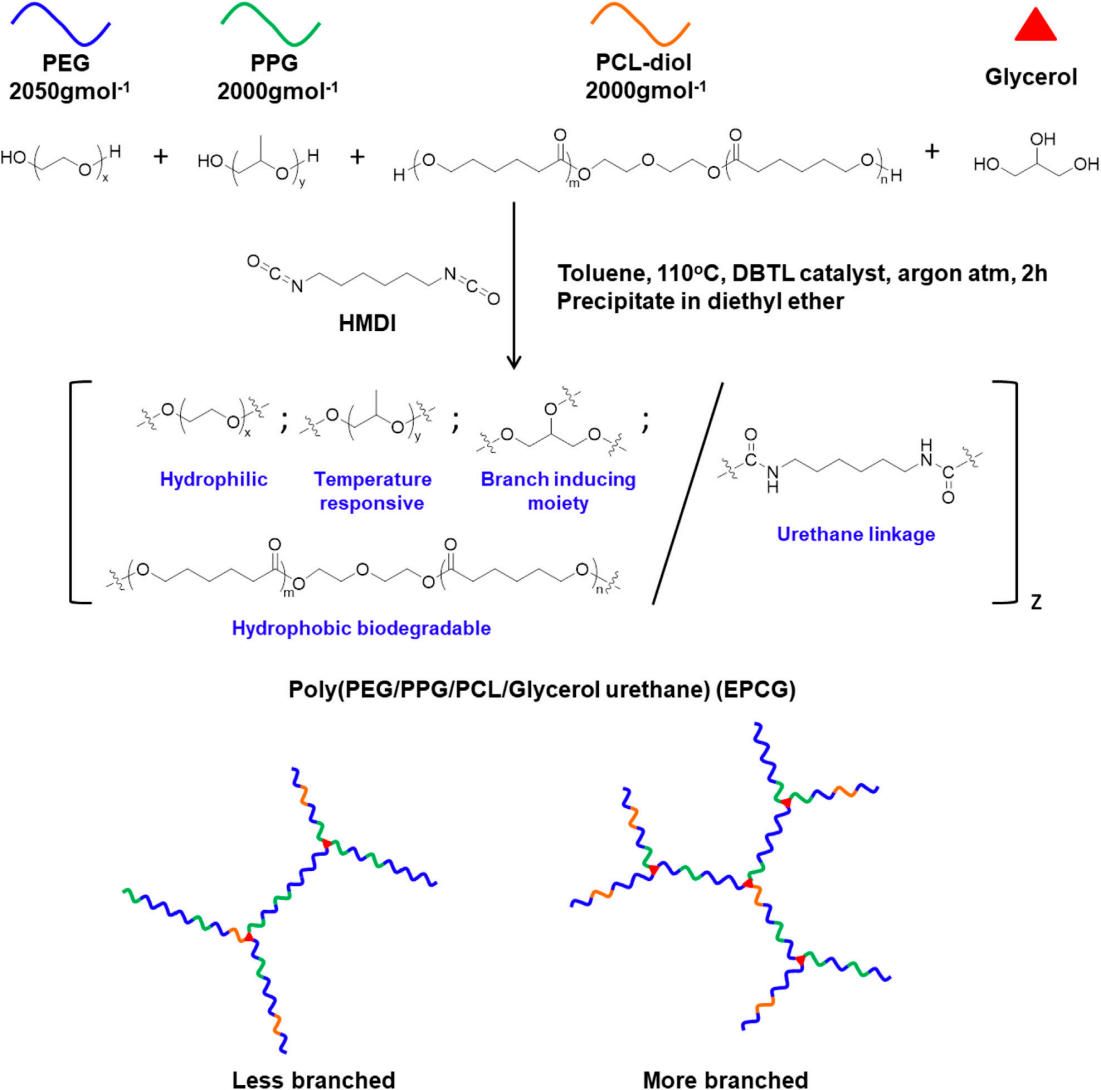
Schematic representation of the synthesis route of poly (PEG/PPG/PCL/Glycerol urethane) (EPCG) branched thermogelling copolymers.

In this study, we designed and synthesised a series of branched amphiphilic polyurethane thermogelling copolymers with varying hydrophobic content and degree of branching by coupling macromonomer-diols, PEG, PPG, PCL-diol, and glycerol with the bis-isocyanate hexamethylene diisocyanate (HMDI) (Figure 1, Supplementary Table S1). Unlike the macromonomer diols, the tri-hydroxylic glycerol molecules function as branching sites, distributed at random throughout the copolymer to form branched architectures (Li et al., 2012). Accordingly, changing the glycerol feed content allows convenient tuning of the degree of branching, with more glycerol producing copolymers with more branches (Figure 1). 2 series of EPCG copolymers were synthesised; EPCG (2:1) refers to the series of copolymers having PEG:PPG molar ratios of 2:1 while EPCG (3:1) refers to the other series of copolymers with molar ratios of PEG:PPG molar ratio of 3:1. In each of the series, the number branches are increased by increasing glycerol feed; G0.25 refers to the sample with the least branches while G0.75 refers to the sample with the most number of branches.

The resultant copolymers were characterised by ^1^H NMR and FTIR. The characteristic resonance peaks of the PEG (δ = 3.64 ppm), PPG (δ = 1.11 ppm), PCL-diol (δ = 2.25–2.30 ppm), and glycerol (δ = 4.05–4.10 ppm) were observed in the respective NMR spectra of the copolymers in CDCl_3_ (δ = 7.25 ppm) (Supplementary Figure S1) and their compositions were calculated from the integrated ratios of the characteristic peaks (Supplementary Table S2). From FTIR, we observed the disappearance of the HMDI isocyanate stretch peak at 2270 cm^−1^ and the appearance of the C=O urethane stretch peak at 1714 cm^−1^ in the spectra of the copolymers (Supplementary Figure S2). These suggest complete reaction between HMDI and the (macro)monomer diols and thus their successful incorporation into the amphiphilic copolymers’ structures.

**FIGURE 2:**
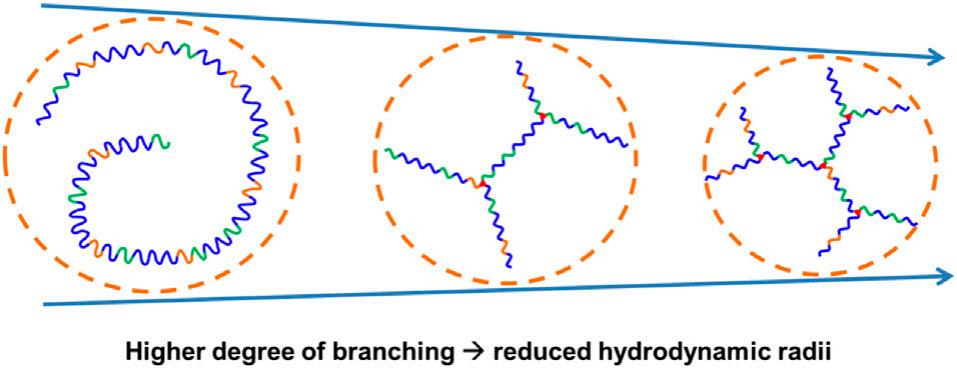
Schematic representation of EPCG copolymers with similar absolute molecular weights but increased degree of branching resulted in more globular and compact architectures with reduced hydrodynamic radii.

The relative and absolute molecular weights of the resultant poly (PEG/PPG/PCL/Glycerol urethane) (EPCG) copolymers were measured by gel permeation chromatography (GPC) and static light scattering (SLS), respectively. Across both EPCG (2:1) and EPCG (3:1) series of increasingly branched copolymers (Table 1), slight increases in retention durations were observed with increased degree of branching. This suggests that the hydrodynamic radii of the EPCG copolymers reduced with higher degree of branching. As expected, determination of their absolute molecular weights by static light scattering (SLS) measurements (Table 1, Supplementary Figure S4) (Wang et al., 2005; Lin et al., 2022) showed larger disparities with the GPC-determined values with higher degrees of branching. Furthermore, while EPC (3:1) G0.5 and EPC (3:1) G0.75 have similar SLS molecular weights (65.4 and 64.9 kDa, respectively), the former was measured to have a larger hydrodynamic radii than the latter (42.2 versus 32.1 kDa). A similar trend is observed between EPC (2:1) G0.5 and EPC (2:1) G0.75. These suggest that higher degree of branching produced copolymers that are increasingly more globular and compact, resulting in them having smaller hydrodynamic radii despite having similar absolute molecular weights as their less branched counterparts (Figure 2). Indeed, quantifying the average number of branches per copolymer and average branch lengths further confirmed that higher glycerol content resulted in more extensive branching with shorter average branch lengths (Table 1). Taken together, these suggest the formation of more globular and compact copolymers with increased polymer branching.

**TABLE 1:** Summary of EPCG copolymers properties.

Sample	M_n_ by GPC (kDa)^a^	PDI	M_w_ by SLS (kDa)^b^	Average number of branches per polymer^c^	Average branch length (kDa)^d^
EPC (3:1) G0.25	74.6	1.94	78.7	2.69	29.2
EPC (3:1) G0.5	42.2	2.01	65.4	3.61	18.1
EPC (3:1) G0.75	32.1	2.07	64.9	5.36	12.1
EPC (2:1) G0.25	68.5	1.56	72.5	1.78	40.8
EPC (2:1) G0.5	46.9	2.05	60.2	3.48	17.3
EPC (2:1) G0.75	37.1	1.74	54.9	5.70	9.64

a GPC measurements were performed using THF as the mobile phase, with molecular weights taken with reference to monodispersed polystyrene standards.

b Static light scattering (SLS) absolute weight average molecular weights of the EPCG copolymers were measured in THF using 5 concentrations (6–10 mg ml^−1^) with toluene as scattering standard. The scattering intensities were plotted against concentration on Debye plots and the absolute molecular weights were obtained from the reciprocals of the intercepts. The value of 0.09 g ml^−1^ was employed as the refractive index increment ( $\frac{dn}{dc}$ ) for the copolymers. This value was obtained by benchmarking using a linear copolymer with similar compositions as the EPCG, copolymers such that this linear copolymer has comparable molecular weights when measured using SLS and GPC (Lin et al., 2022).

c The average number of branches per polymer is obtained by dividing the SLS molecular weight with the average number of glycerol moieties present in the copolymer.

d The average branch length is calculated by dividing SLS molecular weight by average number of branches per polymer.

To gain further insights on how the extent of polymer branching influences the polymer self-assembly process, we further probed the thermodynamics of micellisation using the Arrhenius method (Chan et al., 2018) (Table 2, Supplementary Table S4, Supplementary Figure S5; see Experimental Section for more details). Above the critical micelle concentrations (CMCs), all thermogelling EPCG copolymers form micelles spontaneously in solution (Loh et al., 2009; Chen et al., 2015), as seen by their negative ΔG values (Table 2). In addition, the positive ΔS and ΔH values show conclusively that micellisation is entropically-driven. This is due to large entropy gained from the release of water molecules bound to the hydrophobic PPG polymer segments above the critical micelle temperature (Loh et al., 2010; Yu et al., 1992), leading to preferential association of the hydrophobic polymeric segments which results in micelle formation (Chan et al., 2018). The heat absorbed during PPG dehydration accounts for the endothermic enthalpy change (Loh et al., 2008). From Table 2, we observe that the enthalpy of micellization becomes less endothermic, accompanied by a smaller extent of entropy gain, as the number of branches increased for both EPC (3:1) and EPC (2:1) series. This trend suggests that with higher degree of branching there is less structured water being released from the copolymers during micellisation. Indeed, this can be attributed to the more globular shape and compactness of increasingly-branched copolymers, which limit the accessibility of hydrophobic segments available for desolvation and mutual interaction (Lin et al., 2022). Correspondingly, as the number of branches increased, the overall Gibbs free energy change of micellisation becomes less exergonic (Table 2), suggesting that the process of micellization becomes less spontaneous and the micelles formed become less thermodynamically stable as the polymers become less linear.

**TABLE 2:** Summary of critical micelle concentrations (CMC), thermodynamic quantities of micellization, and micelle sizes of EPCG copolymers.

Sample	CMC (wt%)^a^	ΔH (kJ mol^−1^)^b^	ΔG (kJ mol^−1^)^c^	ΔS (kJ mol^−1^ K^−1^)^d^	Z-average micelle diameters (nm)^e^
EPC (3:1) G0.25	0.0234	72.6	−38.3	0.372	104
EPC (3:1) G0.5	0.0237	67.7	−36.3	0.349	57.9
EPC (3:1) G0.75	0.0250	57.0	−35.5	0.311	44.5
EPC (2:1) G0.25	0.0194	54.8	−39.2	0.315	97.0
EPC (2:1) G0.5	0.0400	55.8	−36.7	0.311	88.3
EPC (2:1) G0.75	0.0231	51.1	−37.8	0.298	54.6

a CMC was measured *via* a dye solubilisation method; dye absorbance was plotted against lg (concentration) and the intersection of the best-fit lines drawn for the unimeric and micellar regions gives the CMC (Supplementary Figure S5B). CMC data shown in this table is obtained at 37°C.

b Enthalpy of micellization is obtained from the gradient of the Arrhenius plot of ln (molar fraction of CMC) against the reciprocal of absolute temperature (Supplementary Figure S5C).

c Gibbs free energy change of micellization is calculated from ln (molar fraction of CMC) using equation 2 at 37°C.

d Entropy of micellization is calculated from equation 4.

e Sols were prepared at 10 mg ml^−1^ at room temperature.

**FIGURE 3:**
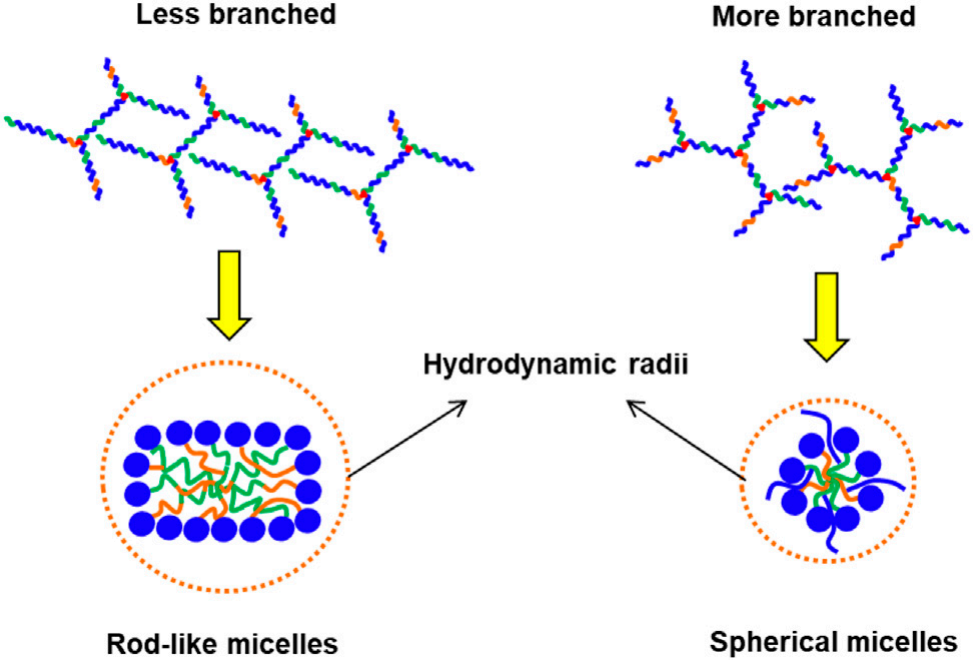
Schematic comparison between the micelles formed by less branched and more branched EPCG copolymers with the former being plausibly more rod-like with larger hydrodynamic radii and the latter being spherical with smaller hydrodynamic radii.

We then determined the influences of polymer branching on the size of the resulting micelles using dynamic light scattering (DLS). As shown in Table 2, more highly-branched polymers result in smaller micelles: the average micelle diameters reduced from 104 to 44.5 nm for the EPC (3:1) series and from 97.0 to 54.6 nm for the EPC (2:1) series as the number of branches increased. This is a consequence of less efficient polymer packing with increased number of branches. It is plausible that relatively linear copolymers can pack closer and form larger micelles while the more branched copolymers experience greater steric hindrance due to their globular geometry and thus form smaller micelles (Figure 3). In addition, the difference in micelle size observed may also be contributed by the difference in micellar shape (Figure 3), with more linear copolymers previously shown to form cylindrical rod-like micelles through small angle X-ray scattering (SAXS) experiments, while the micelles from more branched copolymers being spherical (Lin et al., 2022). The rod-like micelles are likely to contribute to large hydrodynamic radii as compared to their spherical counterparts.

**FIGURE 4:**
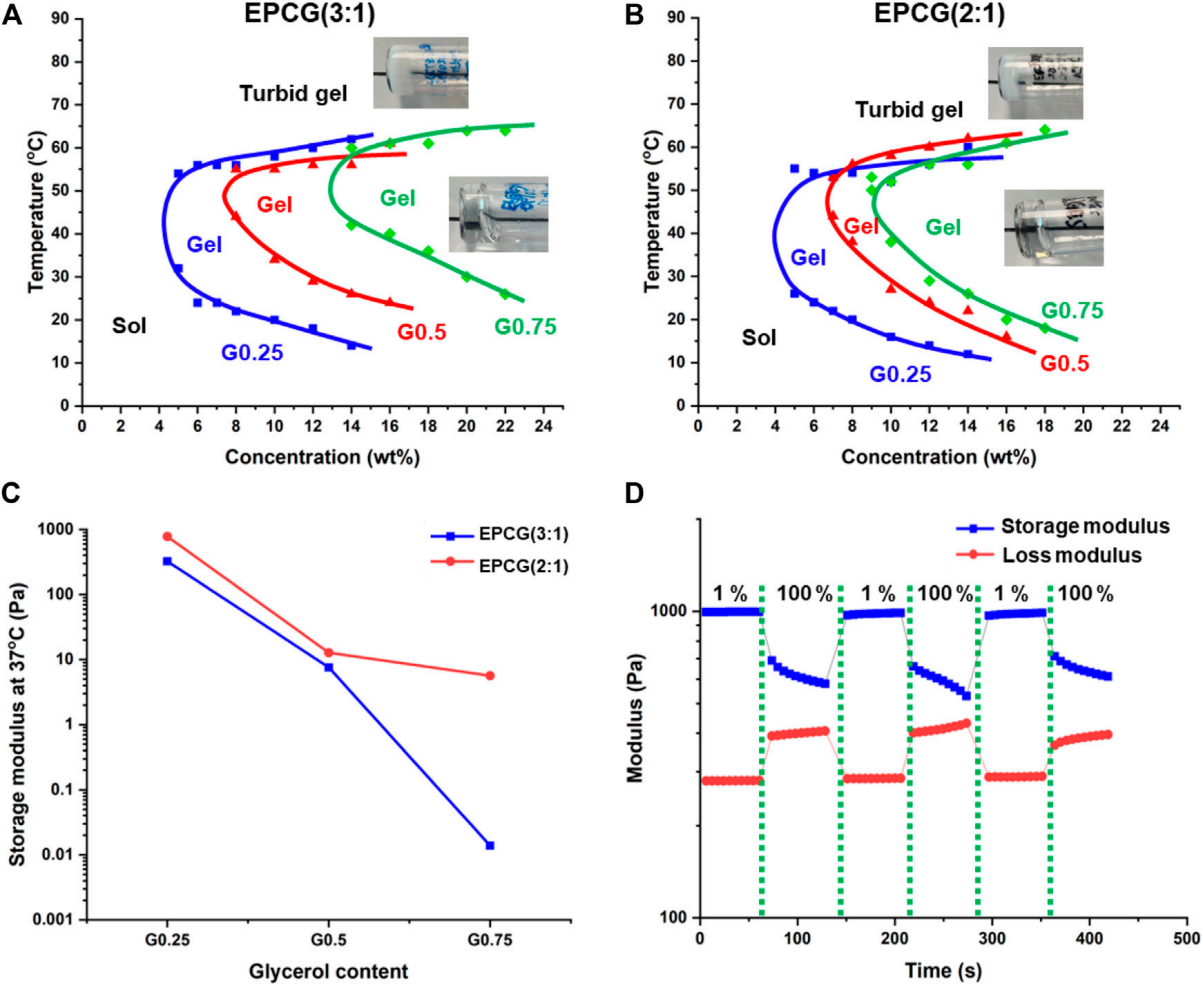
Effect of polymer branching on the temperature-concentration phase diagrams of **(A)** EPCG (3:1) and **(B)** EPCG (2:1) series of thermogels. The pictures are representative images of the gels and turbid gels formed. More images are provided in Supplementary Figure S6. **(C)** Effect of polymer branching on EPCG thermogels’ storage moduli. Measurements were obtained *via* rheology temperature sweep of thermogels at 3°C min^−1^, 1% strain, and 1 Hz. **(D)** Representative rheological measurement of EPCG thermogel [EPC (2:1) G0.25 15 wt%] subjected to repeated low (1%) and high strains (100%) at 1 Hz shows that the thermogel has good shear thinning ability and good recoverability.

All EPCG thermogelling copolymers were found to dissolve in water to form optically clear hydrogels, regardless of the hydrophobic content or degree of polymer branching (Supplementary Figure S6). They also exhibit characteristic temperature responsive gelation by transiting from sol to gel to turbid gel phases as temperature is increased (Figures 4A,B). Gelation is brought about by the partial dehydration of micelles when the thermogel sol is heated such that the micelles aggregate and coalesce into a supramolecular hydrogel matrix (Cally et al., 2018). As such, the gelation temperature is defined as the first temperature where a thermogel formulation is seen to form a non-flowing gel *via* a tube-inversion method. At temperatures much higher than the gelation temperature, the micelles are dehydrated extensively such that they further aggregate together and cause the formation of large light scattering points such that the originally transparent thermogels become opaque (turbid gel) (Liow et al., 2016b). Thus, the temperatures at which the turbid gel first forms demarcate the phase boundary between gel and turbid gel phase (Figures 4A,B).

The critical gelation concentration (CGC) is the minimum copolymer concentration required to achieve the gel phase (Zhang et al., 2021). As branching increases, the CGCs of the EPCG (3:1) polymers increased from 4 wt% to 7.5 wt% to 13 wt% (Figure 4A); while those of the EPCG (2:1) series similarly increasing from 4 wt% to 6.5 wt% to 9wt% (Figure 4B). This is in agreement with more branched copolymers having more globular geometries and thus experiencing greater steric hindrance during their self-assembly. Consequently, the weaker inter-micellar interactions reduce the ease and likelihood of micelle self-assembly into a supramolecular hydrogel matrix. In addition to higher CGC, the gelation temperatures of thermogels at the same concentration also increased with the degree of branching. Together with higher CGCs, an increase in polymer branching is seen to cause the sol-gel phase boundaries to shift rightwards and upwards (Figures 4A,B).

The temperature-responsive gelation behaviours of the EPCG thermogels were also probed *via* rheology. In the representative oscillatory temperature sweep measurement, the loss modulus (G”) was larger in magnitude than the storage modulus (G′) at low temperatures (Supplementary Figure S7). The loss modulus measures the energy loss while the storage modulus measures the energy storage of a viscoelastic material (Grattoni et al., 2001). As such, G″ > than G′ at low temperatures indicate that the thermogel formulation was a viscous fluid. Following the increase in temperature, G′ increases to become equal to G″, with the crossover point taken to be the gelation temperature as the thermogel adopts a more solid-like behaviour than a viscous fluid. Beyond the gelation temperature, G′ becomes significantly higher than G″, suggesting that the thermogel is becoming stiffer and behaving more like an elastic solid.

The storage moduli of the various EPCG formulations at 15 wt% and 37°C (physiological temperature) were compared to investigate the effect of polymer branching on the stiffness of the thermogels. With higher degree of branching, the storage moduli of the thermogels formed decreased significantly (Figure 4C). In the EPCG (3:1) series, the storage moduli decreased by more than 4 decades, while in the EPCG (2:1) series there was a decrease by almost 3 decades. This observation is in line with a higher degree of polymer branching leading to poorer micellar self-assembly during gelation, resulting in thermogels with less rigid supramolecular matrixes.

The EPCG thermogels formed were also demonstrated to have good recoverability after being subjected to high strains, as characteristic of many injectable supramolecular hydrogels (Liu et al., 2018; Ren et al., 2020). They were able to fully regain their storage moduli despite being subjected to repeated cycles of high strains that cause the temporary disruption of the supramolecular matrixes (Figure 4D). This is due to the ability of the intermolecular forces between the micelles, such as hydrophobic association and hydrogen bonding, being able to reform rapidly after the high-strain deformation of supramolecular hydrogel matrix, resulting in no perceivable loss in the mechanical properties of the thermogels. This allows the EPCG branched thermogels to be good candidates as injectable supramolecular hydrogels.

### Stability of EPCG Thermogels to Erosion and Biococompatibility

As supramolecular hydrogels, the EPCG branched thermogels undergo erosion when exposed to external media (Loh, 2018a; Xue et al., 2020a). The external media would gradually dilute the thermogels at the surface and cause them to shed micelles, leading to gradual disassembly of the supramolecular hydrogel matrix. Although these EPCG branched thermogels may also degrade via hydrolysis of the ester bonds present in the PCL components (Ahmed and Discher, 2004; Loh et al., 2007), surface erosion is often the main mechanism of thermogel clearance as the rate of hydrolysis of PCL is much slower as compared to surface dilution of the thermogels (Lee and Gardella, 2000; Woodard and Grunlan, 2018). As the rate of hydrogel erosion directly impacts the release of drug embedded within the hydrogel matrices, we studied the influence of copolymer branching on the rate of surface micelle shedding.

**FIGURE 5:**
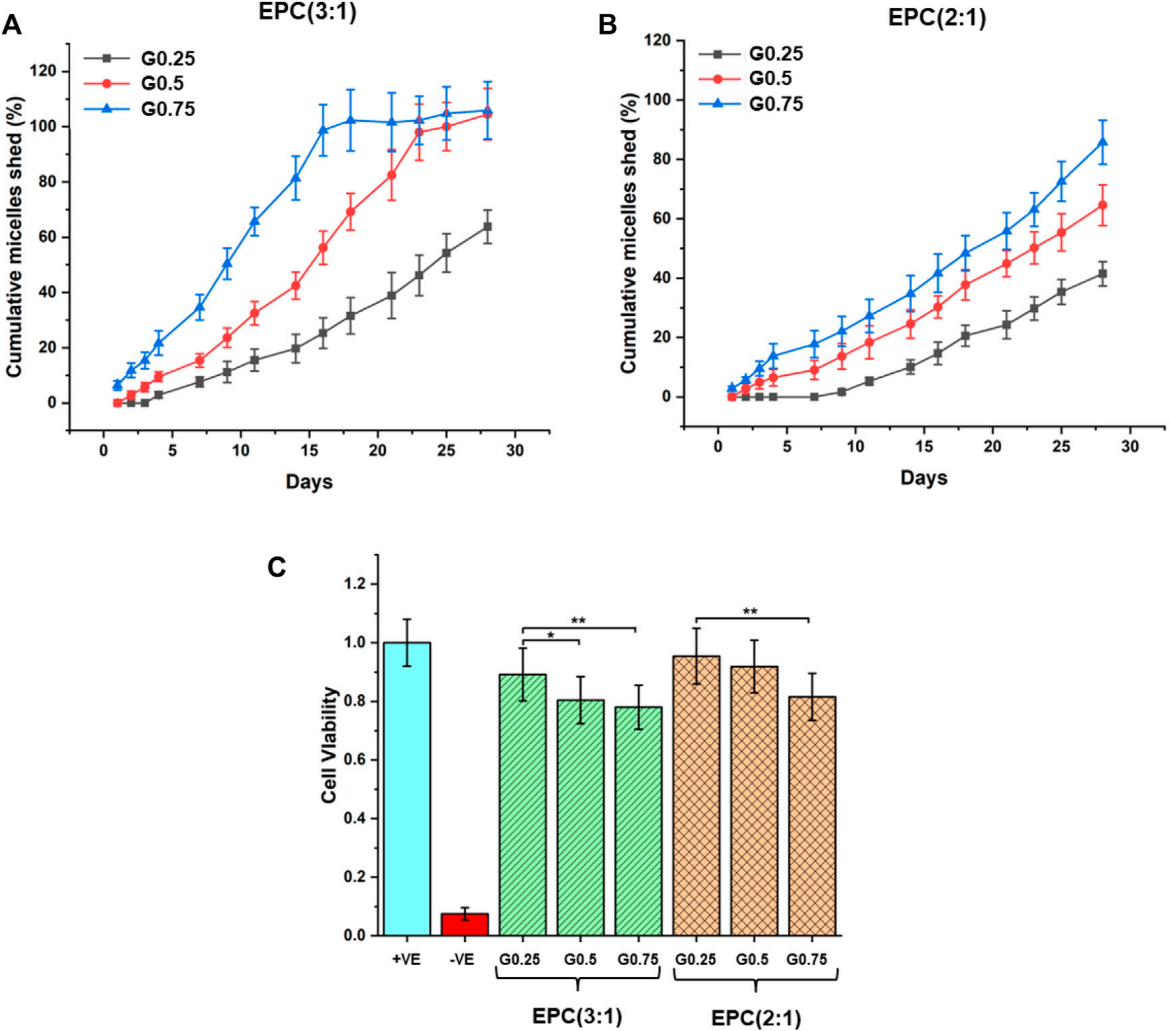
*In-vitro* erosion profiles of **(A)** EPCG (3:1) and **(B)** EPCG (2:1) thermogels based on cumulative amount of micelles shed from bulk thermogels over 4 weeks. **(C)** Biocompatibility of the EPCG thermogels at 15 wt% after incubating them with NIH/3T3 cells for 72 h. In the positive control, cells were incubated with 1x PBS solution while in the negative control, cells were incubated with 1 v/v% sodium dodecyl sulfate (SDS) solution diluted using 1x PBS.

The rates of erosion of the EPCG branched thermogels were measured in an *in-vitro* set-up by quantifying the cumulative amount of micelles shed over time from the thermogels using standard curves correlating dye absorbance to micellar concentration (Supplementary Figure S8) (Xue et al., 2020a). The rates of erosion are observed to accelerate for thermogels formed from increasingly branched copolymers in both the EPCG (3:1) and EPCG (2:1) series (Figures 5A,B). This is consistent with the globular polymers formed from greater branching experiencing more steric hindrance during self-assembly, resulting in weaker inter-micelle interactions that are more susceptible to water penetration and hence erosion. While the erosion profiles of EPCG (3:1) series appears to resemble pseudo-first-order kinetics, the EPCG (2:1) series have erosion profiles that resembles pseudo-zeroth-order kinetics more. This may be attributed to the higher hydrophobic composition of the EPCG (2:1) series which allows this series of thermogels to be more resistant to water penetration and erosion.

The rate of hydrogel erosion has implications on their biocompatibility, as the micelles released can act as surfactants that potentially disrupt cell membranes (le Maire et al., 2000; Neimert-Andersson et al., 2006; Hu et al., 2008; Xue et al., 2020a). Thus, we evaluated the general *in-vitro* biocompatibility of the EPCG branched thermogels by incubating them with NIH/3T3 mouse fibroblast over 72 h (Figure 5C). The results suggest that all EPCG thermogels tested at 15 wt% are biocompatible, with cell viabilities around or greater than 80% with respect to the positive control (cells incubated with 1x PBS), making them suitable candidates for implantation based biomedical applications (Yu et al., 2010; Gori et al., 2020). Despite the general high biocompatibilities across all the EPCG thermogels, cell viability was slightly lowered as the number of branches on the copolymers increased, which is consistent across both the EPCG (3:1) and EPCG (2:1) series (Figure 5C). We attribute this to the more facile erosion from the thermogels formed by more branched copolymers, causing a higher concentration of shed micelles that behave as surfactants and result in increased cellular cytotoxicity.

### Drug Release Studies

**FIGURE 6:**
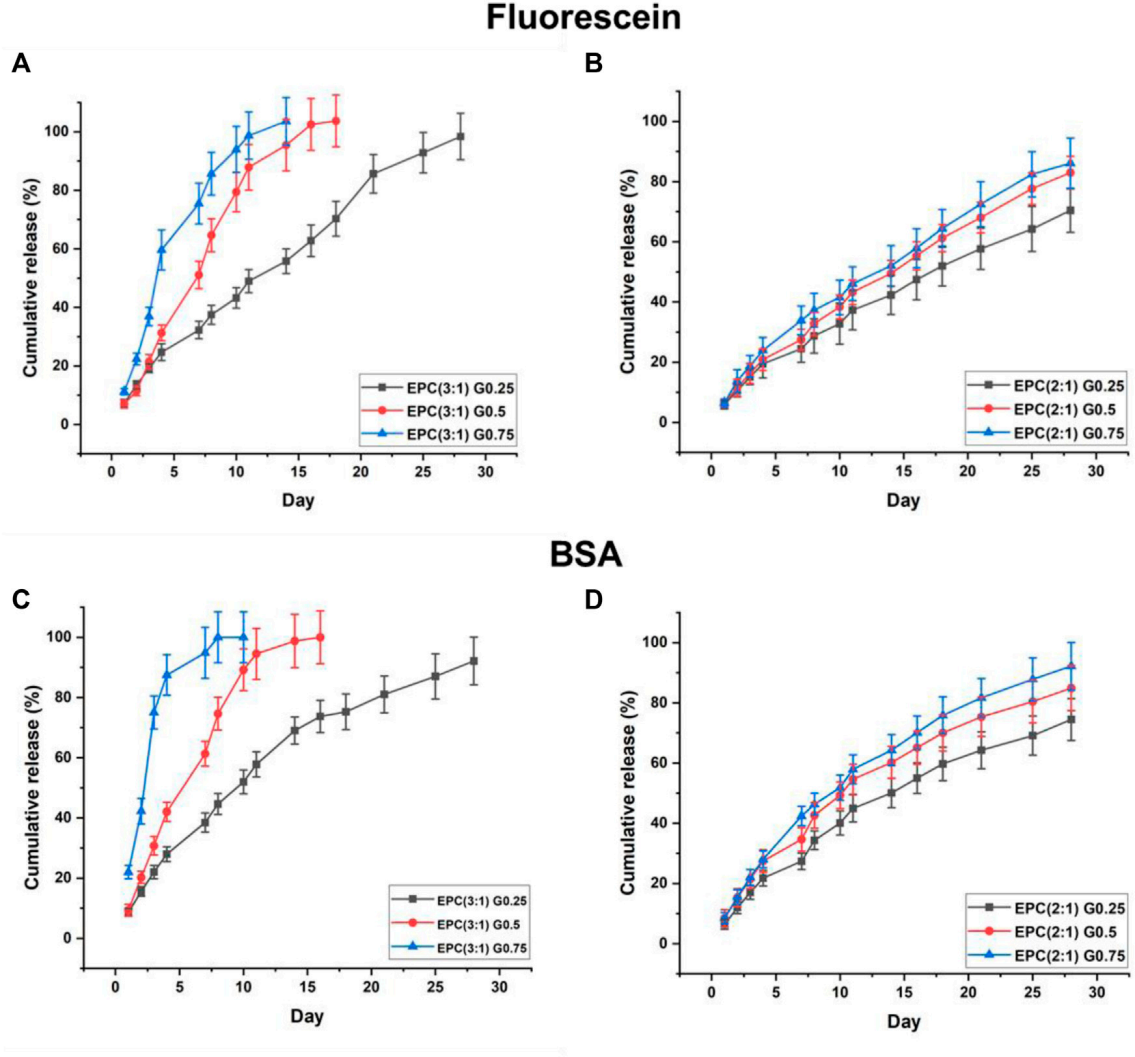
Sustained release of model hydrophobic drug, fluorescein, from **(A)** EPCG(3:1) and **(B)** EPCG(2:1) series of thermogels and sustained release of model hydrophilic protein, BSA, from **(C)** EPCG(3:1) and **(D)** EPCG(2:1) series of thermogels.

The EPCG branched thermogels are able to act as gel depots and encapsulate both hydrophobic drugs and hydrophilic proteins in their supramolecular matrixes by simply mixing the drugs or proteins with the thermogels in their sol phases before applying heat to cause gelation (Loh, 2018a). Together with their shear-thinning property and erodibility, these properties make EPCG thermogels suitable as potential injectable biodegradable platforms for localised sustained delivery of drugs or proteins (Loh, 2018b; Li et al., 2021; Xu et al., 2022). Herein, we investigate the effects of varying polymer branching and hydrophobic composition on the release profiles of fluorescein free acid as the model model hydrophobic drug, and bovine serum albumin (BSA) as the model protein drug, from EPCG branched thermogels (Figure 6).

The rate of fluorescein release increases with the degree of polymer branching (Figure 6A). In the EPCG (3:1) series, the release of fluorescein appears to follow pseudo-zeroth-order kinetics for the thermogel formed by the least branched copolymer (G0.25). In contrast, the rates of fluorescein release resemble pseudo-first-order kinetics for the thermogels formed by the more branched copolymers, G0.5 and G0.75, with the latter having a higher rate of release than the former.

**FIGURE 7:**
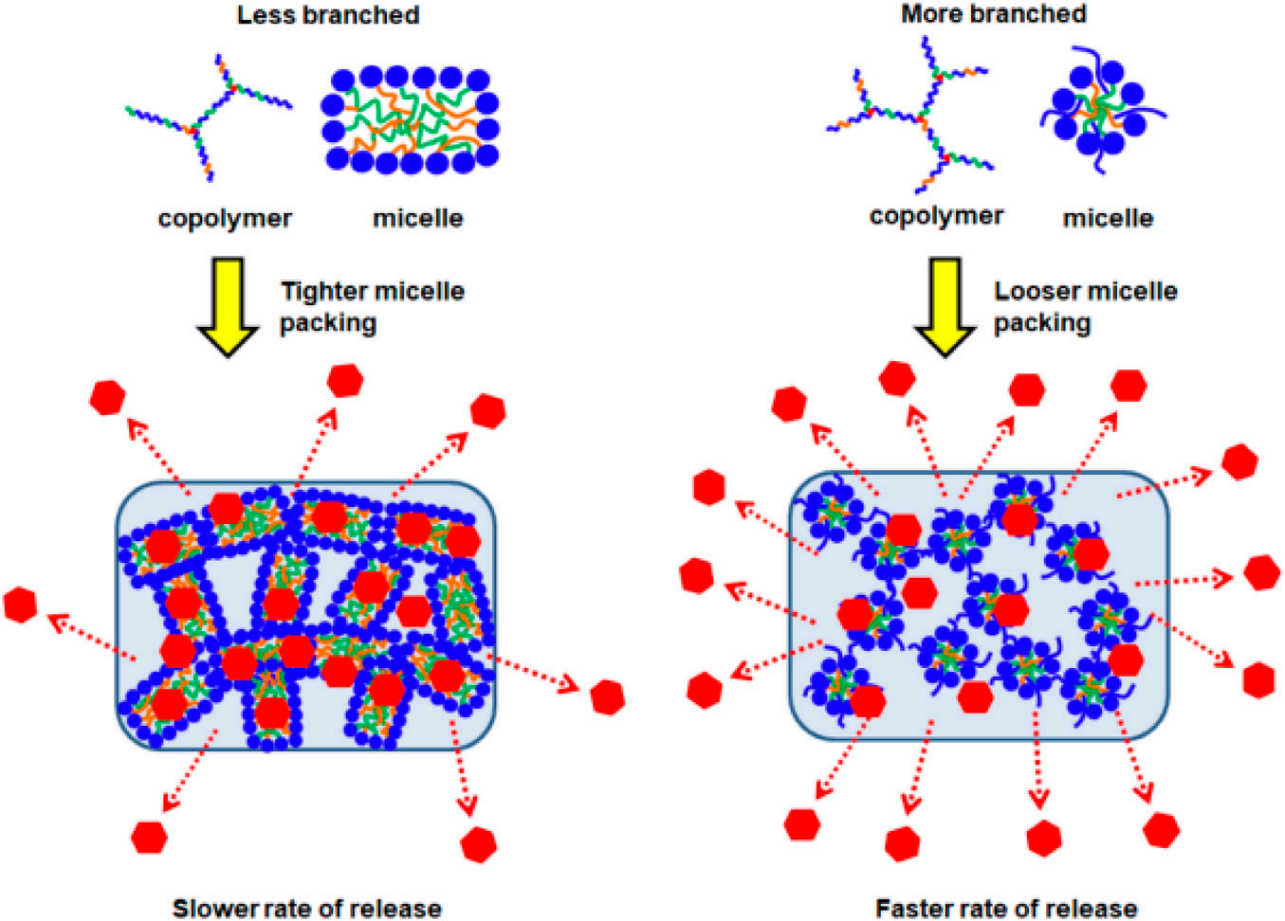
Schematic representation of EPCG copolymers micellar packing into supramolecular hydrogels followed by the release of incorporated drugs; higher degree of branching leads to looser micellar packing and subsequent faster rate of release.

With fewer branches (G0.25), the copolymers form thermodynamically more stable micelles with plausibly good encapsulation of fluorescein in their micellar cores. Furthermore, these micelles are able to pack tightly in the supramolecular hydrogel matrix with higher resistance to water penetration and erosion. Together, these helped suppress fluorescein burst release and also achieve more sustained release (Figure 6A). The relatively linear release profile from EPC (3:1) G0.25 also suggests that the release process is dominated by surface erosion. In contrast, with higher polymer branching, the copolymers form less thermodynamically stable micelles that pack looser in the supramolecular hydrogel matrix. These micelles may be less efficient at encapsulating fluorescein, resulting in the presence of more non-encapsulated fluorescein molecules in the thermogels, and this may have contributed the higher burst releases observed with EPC (3:1) G0.75 > EPC (3:1) G0.5 (Figure 6A). In addition, the release profiles of fluorescein from EPC (3:1) G0.5 and EPC (3:1) G0.75 resembles a pseudo-first-order kinetics, suggesting that the release is diffusion controlled, thus supporting the hypothesis that there are more non-encapsulated fluorescein molecules present in thermogels formed from more branched copolymers (Figure 7).

Similar to the EPCG (3:1) series, increased rate of fluorescein release is observed for thermogels formed with more branched copolymers in the EPCG (2:1) series (Figure 6B). This is expected due to the same differences in polymer geometry and micellar packing. However, unlike the EPCG (3:1) series, the release profiles of fluorescein from EPCG (2:1) series appears to be more linear. This may be attributed to the higher hydrophobic composition of the EPCG (2:1) thermogels, which plausibly allows them to encapsulate fluorescein better and also prolong fluorescein release by eroding slower.

Similar to fluorescein release, the rate of protein release increases with the degree of polymer branching in both EPCG (3:1) and EPCG (2:1) series (Figures 6C,D). However, the release profiles of BSA from all EPCG thermogels appear to be more diffusion controlled with pseudo-first-order kinetics with more prominent curvatures, as compared to being more surface erosion-controlled (Varnier et al., 2018). Due to the hydrophilic nature of BSA, they are more likely to be entrapped in the supramolecular matrix pores (Kiene et al., 2018) instead of the hydrophobic micellar pores like fluorescein. As such, the release of BSA would be reliant upon the reptation of the protein molecules through the physical barriers presented by the supramolecular matrix (Hettiaratchi et al., 2018). The faster rate of release from increasingly branched polymers can thus be ascribed to the looser micellar packing that would potentially reduce the physical barriers and allow for higher rates of BSA diffusion. Moreover, the looser micellar packing is also correlated with faster water penetration and erosion, which would also contribute to faster BSA release.

Lastly, it appears that while an increase in copolymer branching caused significantly higher rates of BSA diffusion out of the EPCG (3:1) thermogels, a similar increase in the number of branches did not lead to significantly higher diffusion rates in the EPCG (2:1) thermogels. This may be because the more hydrophobic EPCG (2:1) thermogels are more resistant to water penetration erosion, such their supramolecular matrixes remain intact for a longer period of time as compared to the EPCG (3:1) series. Therefore, the EPCG (2:1) thermogels have more gradual release profiles as compared to the more hydrophilic EPC (3:1) thermogels.

## Conclusion

In conclusion, we have synthesised a family of amphiphilic copolymers bearing different degrees of branching that are able to form transparent thermogels, by introducing the triol, glycerol as a branching point in isocyanate-hydroxyl polyaddition reactions. While the release profiles of drugs and proteins from thermogels are usually controlled by varying molecular weight and copolymer composition, we have demonstrated that the degree of branching on the copolymer can also be employed to systematically tune the release profiles. More branches on the copolymers cause them to become increasingly globular, which enforces greater steric hindrance during self-assembly to form less tightly-associated micelles and hydrogels. This subsequently affected the bulk thermogel erosion rates, and a correspondingly higher rate of release of both fluorescein and BSA with an increase in copolymer branching. By demonstrating the important influences of the hitherto-overlooked parameter of polymer branching on the rates of drug release, this work widens the possibilities of advanced biomaterial design for bespoke localised sustained-drug release therapy.

## Data Availability

The raw data supporting the conclusion of this article will be made available by the authors, without undue reservation.
