# Supplementary material for: Branched PCL-Based Thermogelling Copolymers: Controlling Polymer Architecture to Tune Drug Release Profiles
Source: Front Bioeng Biotechnol. 2022 Mar 30;10:864372. doi: 10.3389/fbioe.2022.864372 (PMC9006874; doi:10.3389/fbioe.2022.864372)
Supplement: Supplementary file 1 [file DataSheet1.docx]

Supplementary Material

Branched PCL-based thermogelling copolymers: Controlling Polymer Architecture to Tune Drug Release Profiles

Qianyu Lin^1,2^, Valerie Ow^2^, Yi Jian Boo^2^, Vincent T.A. Teo^3^, Joey H. M. Wong^2^, Rebekah P.T. Tan^2^, Kun Xue^2^, Jason Y.C. Lim^2,4^*, Xian Jun Loh^2,3,4^*

^1^ NUS Graduate School for Integrative Sciences and Engineering, National University of Singapore (NUS), 21 Lower Kent Ridge Rd, 119077, Singapore

^2^Institute of Materials Research and Engineering (IMRE), Agency for Science, Technology and Research (A*STAR), 2 Fusionopolis Way, 138634, Singapore

^3^School of Materials Science and Engineering, Nanyang Technological University, 50 Nanyang Avenue, #01-30 General Office, Block N4.1, 639798, Singapore

^4^Department of Materials Science and Engineering, National University of Singapore (NUS), 9 Engineering Drive 1, 117576, Singapore

*** Correspondence:**Asst. Prof. Jason Y. C. Lim
[jason_lim@imre.a-star.edu.sg](mailto:jason_lim@imre.a-star.edu.sg)

Prof. Xian Jun Loh

[lohxj@imre.a-star.edu.sg](mailto:lohxj@imre.a-star.edu.sg)

# Materials

# PEG with M_n_ 2,050 g mol^−1^, PPG with M_n_ 2,000 g mol^−1^, PCL-diol with M_n_ 2,000 g mol^−1^, glycerol, 1,6-hexamethylene diisocyanate (HMDI) (98%), dibutyltin dilaurate (95%), 1,6-diphenyl-1,3,5-hexatriene (DPH) (98%), anhydrous toluene, and fluorescein free acid were purchased from Sigma-Aldrich (St. Louis, Missouri, USA). Diethyl ether and ethanol absolute were purchased from VWR Chemicals (Radnor, Pennsylvania, United States). Isopropyl alcohol of CMOS grade was purchased from J.T. Baker, Avantor (Radnor, Pennsylvania, United States). Pre-wetted regenerated cellulose dialysis membrane with molecular weight cut-off of 3500Da (Spectra/Por^®^ 6) and tetrahydrofuran (THF) of high purity liquid chromatography (HPLC) were purchased from Thermo Fisher Scientific (Carlsbad, CA, USA). Chloroform-D (D, 99.8%) was purchased from Cambridge Isotope Laboratories Incorporation (Massachusetts, MA, USA). Potassium bromide for infra-red (IR) spectrocopy Uvasol ® was purchased from Merck (Darmstadt, Germany). NIH3T3 cells were purchased form American Type Culture Collection (Manassas, Virginia, USA). Gibco™ Dulbecco's modified Eagle's medium (DMEM), fetal bovine serum (FBS), penicillin-streptomycin (PS), Gibco™ sterile phosphate buffered saline 1x (PBS), Gibco™ 0.25% trypsin-EDTA 1x, and Pierce^TM^ BCA Protein Assay Kit were purchased from Thermo Fisher Scientific (Carlsbad, CA, USA). Sodium dodecyl sulfate (SDS) solution (10% w/v) of molecular biology grade and Cell-Titer Blue assay were purchased from Promega (Madison, Wisconsin, USA). Dimethyl sulfoxide (DMSO) for molecular biology was purchased from Sigma-Aldrich. 8 µm polycarbonate transwells fitted for 24-well plates were purchased from Corning (New York, USA). All chemicals and materials were used as received unless otherwise stated.

**2. Experimental methods**

*Synthesis of branched poly(PEG/PPG/PCL/glycerol urethane)s (EPCGs).* PEG (M_n_ 2,050 g mol^−1^), PPG (M_n_ 2,000 g mol^−1^), PCL-diol (M_n_ 2,000 g mol^-1^), and glycerol were weighed out into 250 mL round bottom flasks according to the amounts detailed in **Table S1**. The (macro)monomers were dissolved in 15 mL of anhydrous toluene at 60 ^o^C and 2 rounds of azeotropic distillation were performed to dry the (macro)monomers. A viscous slurry was obtained after the azeotropic distillation. The slurry was further dried under high vacuum at 110 ^o^C for 30 mins. Thereafter, inert dry argon was introduced into the reaction flask and 30mL anhydrous toluene was added to dissolve the (macro)monomers at 110 ^o^C. 5 uL of dibutyltin dilaurate (DBTL) catalyst was added followed by hexamethylene diisocyanate (HMDI). The polymerisations occurred over 2 h before the reactions were quenched with 5 mL of absolute ethanol. The reaction mixtures were precipitated into anhydrous diethyl ether to obtain the crude copolymers. The crude copolymers were further purified by dialysis. 5 g of crude copolymer was dissolved in 50 mL of CMOS-grade isopropyl alcohol at 60 ^o^C and filled into regenerated cellulose dialysis tubing with 3500 Da molecular weight cut-off. Dialysis was performed against 2 L deionised water for 3 days with 2 changes of water per day. The dialysed copolymer solution was frozen and lyophilised to obtain the purified polymer (typical yield = 4.5 g, ≈ 90 %).

*Poly(PEG/PPG/PCL/glycerol urethane) (EPCG) copolymer characterisations*. Apparent molecular weights of the copolymers were determined by Gel Permeation Chromatography (GPC) using Agilent 1260 Infinity II. The GPC system was equipped with 1260 Vial-sampler, 1260 Iso-Pump, 1260 Refractive Index Detector (RID), and an Agilent PLgel 5 µm MIXED-D column with molecular weight range of 200 Da to 400,000 Da. The system was circulated with THF of HPLC grade at 1 mL min^-1^ and 40 ^o^C. Monodispersed polystyrene standards with M_p_ of 162, 370, 935, 1250, 3050, 6660, 12980, 27060, 56600, 91450, 217100, 36400 g mol^-1^ were used to obtain a calibration curve. Copolymers samples were injected at 5 mg mL^-1^ and 20 µL for GPC measurements. The GPC plots against retention of EPCG copolymers are provided in **Figure S3**.

Due to the branched nature of the EPCG copolymers, GPC is insufficient to characterise the molecular weights as it is calibrated based on linear standards. The absolute molecular weights of the copolymers were thus measured by employing static light scattering (SLS) using Zetasizer (Nano ZS (Malvern Instruments, Southborough, Massachusetts, USA). Absolute molecular weights are calculated using the following Rayleigh equation (Equation 1):

(1)

$$\frac{\mathrm{Kc}}{R_{90}}= \frac{1}{M_{w}}+2A_{2}C$$

where $K=\frac{4\pi^{2}n^{2}{(\frac{\mathrm{dn}}{\mathrm{dc}})}^{2}}{\lambda^{4}N_{A}}$

K is the Debye constant; c is sample concentration; R_90_ is the Rayleigh ratio at 90^o^; M_w_ is the weight average molecular weight; A_2_ is the second virial coefficient; $\frac{dn}{dc}$ is the refractive index increment; λ is wavelength of light in vacuum; N_A_ is Avagadro’s number. The scattering constant used was toluene (refractive index = 1.496, Rayleigh ratio = 1.35 * 10^-5^ cm^-1^) and the refractive index increment ($\frac{dn}{dc})$ was set to 0.09 mL g^-1^. $(\frac{dn}{dc})$ was estimated such that the GPC molecular weight of a linear poly(PEG/PPG/PCL urethane) of similar composition to the branched EPCGs was similar to its SLS molecular weight.^38^ (For comparison, the value of 0.13 mL g^-1^ was given for PEG 4000 g mol^-1^ and 6000 g mol^-1^ dissolved in water.^67^) The small molecule model was employed, thus no shape correction was performed. The absolute molecular weights are given by the reciprocal of the intercept of the best-fit line of the Debye plots (reduced scattering intensity (Kc/R_90_) plotted against the concentration of copolymers in THF with solvent refractive index = 1.409) (**Figure S4**).

^1^H nuclear magnetic resonance (NMR) spectra were recorded using a JEOL 500 MHz NMR spectrometer (Tokyo, Japan) at room temperature and chemical shifts were referred to the solvent peak of deuterated chloroform (CDCl_3_, δ = 7.25 ppm). The acquisition time was 4.37 seconds, pulse repetition time was 9.37 seconds, pulse width was 90^o^, and 64 scans were performed per sample (**Figure S1**).

Fourier transform infrared (FTIR) spectra of the copolymers were recorded on a FTIR spectrometer (Perkin Elmer Spectrum 2000). 64 scans were signal-averaged with a resolution of 4 cm^-1^ from 4000 cm^-1^ to 300 cm^-1^. The EPCG copolymers are dissolved in THF at 5 mg mL^-1^. 20 μL of each copolymer solution was dripped onto individual potassium bromide (KBr) pellets that were pressed using a manual hydraulic press (Specac, 15011). The pellets were air dried overnight before being scanned by the spectrometer. The FTIR spectrums of the macromonomers, glycerol, HMDI, and EPCG copolymers are also included in **Figure S2**. For PEG, 5 mg of the solid was crushed and mixed with KBr before being pressed into a pellet. For PPG, glycerol, and HMDI, about 5 mg of each ingredient were spread out on individual KBr pellets.

*Critical micelle concentration (CMC) determination by dye solubilisation method*. Each EPCG copolymer was dissolved at 1 wt% in 10 mL of deioinsed water overnight before being diluted into 10 concentrations between 0.001 wt% and 1 wt% at 4 mL per concentration. 40 µL of 0.6 mM DPH solution (in methanol) was added to each sample concentration. The solutions were then kept in a dark cabinet overnight to allow the methanol to evaporate and the DPH dye to become encapsulated in the micellar cores. The absorbance spectra of DPH were recorded using a UV-VIS Spectrophotometer (Shimadzu, UV-2501 PC, Kyoto, Japan) between 320 nm and 420 nm (**Figure S5A**). The difference in absorbance at 376 nm and 400 nm (A_376_ – A_400_) were plotted against log(concentration) and 2 regimes can be clearly observed. The regime with low absorbance values is the unimeric regime where no micelles have formed, while the regime with increasing absorbance values is the micellar regime. Each regime is fitted with a best-fit line and extrapolated. The intersection of the best-fit lines gives the CMC value (**Figure S5B**). CMC values of each copolymer were found at 4 temperatures (15 ^o^C, 25 ^o^C, 37 ^o^C, 45 ^o^C) by equilibrating samples for 20 min in water baths of the stated temperatures before recording their absorbance spectra (**Table S4**).

*Determination of thermodynamic quantities of micellization.* Assuming a closed association of unimers into micelles,^19^ the thermodynamic parameters related to the micellization process may be calculated based on the following equations. Free energy of micellization, ΔG, may be calculated by Equation 2 (**Table 2**):

(2)

$$\Delta G=RTln(X_{\mathrm{CMC}})$$

where R is the ideal gas constant, T is the temperature in K, and X_CMC_ is the CMC in mole fractions of copolymer in the aqueous solution at temperature T. Negative ΔG values indicate the spontaneous formation of thermodynamically stable micelles^39^. Standard enthalpy (ΔH) of micellization may be calculated from the gradient of an Arrhenius plot of ln(X_CMC_) against T^-1^ using Equation 3 (**Figure S5C**). The entropy (ΔS) of micellization (**Table 2**) is next calculated from Equation 4:

$\Delta H=R\left( \frac{\mathrm{dln}\left( X_{\mathrm{CMC}} \right)}{dT^{-1}} \right)$

(3)

$\Delta S=\frac{\Delta H-\Delta G}{T}$

(4)

*EPCG micelle hydrodynamic size.* The EPCG copolymers were dissolved at 1 wt% (1 mL) in deioinsed water at 4 ^o^C overnight and allowed to warm to room temperature for 4 h before the hydrodynamic sizes of their micelles were measured by dynamic light scattering (DLS) using Zetasizer Nano ZS (Malvern Instruments, Southborough, Massachusetts, USA) equipped with a laser light wavelength of 633 nm and operated at 173° scattering angle. The z-average hydrodynamic radius based on intensity-size distributions were obtained (**Table 2**).

*Preparation of poly(PEG/PPG/PCL/glycerol urethane) (EPCG) gels*. EPCG themogels were prepared by dissolving dry purified copolymers in deionised water at 4 ^o^C overnight to obtain homogenous sols and warming the sols above their gelation temperatures.

*Sol−Gel-Turbid Gel phase diagrams by tube inversion method.* Gel samples of 1mL each with concentrations ranging from 4 wt% to 20 wt% were prepared in glass vials and heated from 4 ^o^C to 70 ^o^C at 2 ^o^C intervals. The gel samples were immersed for 5 min at each temperature and inverted for 30 s. The gelation temperature (sol-gel phase boundary) is defined as the lowest temperature at which a non-flowing gel is formed. On the other hand, the temperature at which the gel sample first turned completely white is demarcates gel to turbid gel phase transition boundary.

*Rheological characterizations of the EPCG thermogels.* Rheological measurements were performed using a TA Instruments Discovery DHR-3 hybrid rheometer (New Castle, Delaware, USA) fitted with 40 mm flat-plate geometry and a temperature-controlled peltier base plate. Temperature sweep measurements were performed in the range of 10 ^o^C to 40 ^o^C at a heating rate of 3 ^o^C min^-1^ with strain fixed at 1% and frequency of 1 Hz (**Figure S7**). The temperature at which storage modulus intersects the loss modulus is the gelation temperature. The storage modulus (measuring the stiffness of the gel) and complex viscosity of the gel at 37 ^o^C were also recorded. Oscillatory time sweep experiments where the gels were subjected to repeated cycles of low strain (1 %) and high strain (1000 %) for 60 s each at 37 ^o^C were performed to investigate their abilities to recover their viscoelastic properties after being subjected to repeated disruptions in their supramolecular matrixes.

*Culturing of NIH/3T3 cell line.* NIH/3T3 (CRL-1658^™^) mouse embryo fibroblast cell line was proliferated in DMEM supplemented with 10 % v/v FBS and 1 % v/v penicillin streptomycin in T75 culture flasks at 37 ^o^C and 5 % CO_2_ (Sanyo MCO-18AIC(UV) Incubator, Marshall Scientific, Hampton, New Hampshire, USA) and were harvested for seeding when confluent. The cell layer was detached using 2 mL of 0.05% Trypsin-EDTA and diluted to 50,000 cells mL^-1^ before seeding at 1 mL per well in 24-well plates.

*Cell viability of EPCG thermogels.* NIH/3T3 cells were used to validate the cell viability of EPCG thermogels. Each EPCG thermogel was dissolved at 15 wt% in sterile PBS solution for 24 h at 4 ^o^C to obtain homogeneous sols. The EPCG sols were loaded into individual 1 mL syringe while ensuring no bubbles were present in the syringe. The sols were aliquoted into transwells (polycarbonate membrane with 8 µm pores fitted for 24-well plate) at a volume of 200 µL per transwell in triplicates. The sols were warmed at 37 ^o^C in an incubator for 10 mins before being transferred into the 24-well plate with the NIH/3T3 cells. The thermogels were then incubated with the NIH/3T3 cells for 72 h at 37 ^o^C and 5% CO_2_. For positive control, 100 uL PBS solution was added to the transwell and incubated with the cells. While for negative control, 100 uL of 1 v/v% SDS solution was added in the transwell and incubated with the cells. After 72 h of incubation, all transwells were removed and the cell culture media in all wells were replaced with fresh 1 mL DMEM per well. 100 µL of CellTiter-Blue^®^ reagent was added to each well and incubated for 2 hours. The viable cells present would convert the redox dye (resazurin) into a fluorescent end product (resorufin). 6 replicates of 100 µL solution from each EPCG thermogel were plated into a black 96-well plate and their fluorescent intensities at 590 nm (excitation at 560 nm) were recorded on by a microplate reader (Infinite M200, Tecan, Switzerland). Cell viabilities were obtained by comparing the fluorescent intensities to the positive control. Standard deviation was calculated by one-way ANOVA with significance level of p < 0.05.

*In-vitro gel erosion study*. The rates of erosion of the EPCG thermogels were quantified *in-vitro* by tracking the cumulative amount of micelles shed over 4 weeks via a dye solubilisation method. First, a standard curve correlating micelle concentration to dye absorbance was made for each EPCG copolymer. Each EPCG copolymer was dissolved at 2 wt% (2 mL) in deionised water at 4 ^o^C overnight before being diluted to 10 concentrations between 0.01 wt% to 2 wt% of 200 µL per concentration and plated into a transparent 96-well plate. 10 µL of 0.6 mM DPH (dissolved in methanol) was added per well. The solutions were aired in a dark cabinet overnight to allow the methanol to evaporate before the absorbance spectrum of DPH between 320 nm and 420 nm was recorded using a plate reader (Infinite M200, Tecan, Switzerland). The absorbance (376 nm – 400 nm) were plotted against concentration for each EPCG copolymer and a best-fit quadratic line was fitted (**Figure S8**). Next, EPCG gels were prepared at 15 wt% (1 mL) and loaded in triplicates into transwells (8.0 µm polycarbonate membrane, 6.5 mm insert, 24-well plate, Corning^®^) at 200 µL per well. The exterior well was filled with 0.5 mL of deionised water each and the gels were incubated at 37 ^o^C for 4 weeks. Micelles shed from the gels passes through the transwell membrane and are collected in the exterior well. These micellar solutions were collected at regular intervals and replaced with fresh 0.5 mL deionised water each time. The shed micelle solutions were plated at 100 µL in transparent 96-well plates in triplicates for each EPCG thermogel sample and 10 µL of 0.6 mM DPH methanol solution was added to each well. Methanol was allowed to evaporate overnight from the solutions before the absorbance spectrums were recorded from 320 nm to 420 nm. The averaged absorbance values (376 nm – 400 nm) from the triplicates were used to calculate the corresponding shed micelle concentrations by utilising the best-fit equations found previously. The concentration of shed micelles was converted to mass of polymer and finally the cumulative percentage polymer mass loss was plotted over time for each EPCG gel sample.

*Quantification of released fluorescein from EPCG thermogels.* Fluorescein solutions of 4 mg mL^-1^ in tetrahydrofuran (THF) were prepared. The EPCG thermogels (15 wt%) were prepared beforehand by dissolving 0.15 g of dry copolymers in deionised water at 4 ^o^C overnight. 40 uL of fluorescein solution was spiked into each EPCG thermogel (1 mL) and homogenised such that the fluorescein final concentration in EPCG thermogel was 0.16 g L^-1^. The fluorescein loaded EPCG thermogels were loaded in triplicates of 200 uL into transwells (8.0 µm polycarbonate membrane, 6.5 mm insert, 24-well plate, Corning^®^) and the external well was filled with 0.5 mL of deionised water. The fluorescein released was thus collected in the external well. The released fluorescein was collected at regular intervals and replaced with fresh 0.5 mL of deionised water. The collected supernatants were kept at −20 °C for quantification after 4 weeks. To relate the fluorescein absorbance to its concentration, the following Beer-Lambert Law (Equation 5) was applied:

(5)

𝜀𝑙=𝐴/𝑐

where A is the measured absorbance of sample solution at specified wavelength, c is molar concentration of solution, 𝜀 is molar absorption coefficient, and 𝑙 is the optical path length. A standard sample was prepared by spiking 40 uL of fluorescein THF solution into 1 mL of deionised water. Its absorbance spectrum was obtained by a microplate reader (Infinite M200, Tecan, Switzerland) and an absorbance maximum at 448 nm was observed with absorbance value of 0.91 (**Figure S9**). The value of c was calculated to be 4.81*10^-3^ mol L^-1^, and as such the constant 𝜀𝑙 was calculated to be 189 mol^-1^ L. The absorbance values of the collected supernatants were measured at 448 nm on the plate reader and concentration of fluorescein was computed by applying Beer-Lambert Law with the above constants. The concentration of released fluorescein was converted to cumulative mass of fluorescein released and plotted over time to investigate the fluorescein release kinetics.

*Quantification of released BSA from EPCG thermogels.* Pierce^TM^ BCA Protein Assay Kit was used as per its instructions. A calibration curve based on BSA at 562 nm was obtained by serial dilution of BSA (1mg mL^-1^) using 1x PBS. A quadratic polynomial trend line was used to fit the calibration curve (**Figure S10**). 40 ug mL^-1^ of BSA solution was prepared in 1x PBS and 0.15 g of each EPCG copolymer was separately dissolved in 1 mL of BSA solution at 4^o^C overnight to form 15 wt% thermogels. The EPCG thermogels loaded with BSA were loaded into 1 mL syringes and aliquoted in triplicates into transwells (8.0 µm polycarbonate membrane, 6.5 mm insert, 24-well plate, Corning^®^) and the exterior well was filled with 0.5 mL of 1x PBS; the set-up was incubated at 37^o^C throughout the course of the experiment. The released BSA was collected in the exterior well solution at regular intervals over 4 weeks and each time the exterior well was replaced with fresh 1x PBS solution. The collected BSA supernatant from each EPCG thermogel at each time-point were plated in triplicates and their concentrations were determined using the BCA assay and calibration curve. The BSA concentrations were converted to cumulative mass of BSA release and plotted against time.

## 3. Supplementary Tables

**Table S1**: Feed ratios during the synthesis of the EPCG (2:1) and EPCG(3:1) copolymers and resultant copolymer composition by ^1^H NMR.

| Samples | PEG-2kDa  (g) | PPG-2kDa  (g) | PCL-diol-2kDa  (g) | Glycerol (mg) | HMDI (ml) | Final PEG:PPG:PCL:Glycerol molar ratio in copolymer by ^1^H NMR |
| --- | --- | --- | --- | --- | --- | --- |
| EPC(3:1)  G0.25 | 3.75 | 1.25 | 0.050 | 12.5 | 0.419 | 3.48: 1.00 : 0.0415: 0.315 |
| EPC(3:1)  G0.5 |  |  |  | 25.0 | 0.441 | 3.55: 1.00: 0.0419: 0.518 |
| EPC(3:1)  G0.75 |  |  |  | 37.5 | 0.462 | 3.52: 1.00: 0.0413: 0.768 |
| EPC(2:1)  G0.25 | 3.33 | 1.67 | 0.050 | 12.5 | 0.420 | 2.30: 1.00: 0.0358 : 0.166 |
| EPC(2:1)  G0.5 |  |  |  | 25.0 | 0.441 | 2.50: 1.00: 0.0308: 0.416 |
| EPC(2:1)  G0.75 |  |  |  | 37.5 | 0.463 | 2.53: 1.00: 0.0304: 0.752 |

**Table S2**. Representative composition calculation of EPCG copolymer (EPC(2:1) G0.75) from ^1^HNMR integration ratios.

| Component | PEG | PPG | PCL | Glycerol |
| --- | --- | --- | --- | --- |
| Corresponding NMR peak label | a | b | k | f |
| Mass of (macro)monomer (Da) | 2050 | 2000 | 2000 | 92.1 |
| Mass of repeating unit (Da) | 44 | 58 | 114 | 92.1 |
| No. of repeating units per (macro)monomer | 46.2 | 34.2 | 16.5 | 1.00 |
| No. of contributing protons per repeating unit | 4 | 3 | 2 | 4 |
| No. of protons contributing to peak on NMR | 185 | 103 | 32.9 | 4.00 |
| NMR integration ratios of respective characteristic peaks | 154 | 33.8 | 0.330 | 0.990 |
| Molar ratio of (macro)monomer in copolymer normalised to PPG | 2.53 | 1.00 | 0.0304 | 0.752 |

**Table S3.** Summary of integration integrals and final compositions of EPCG copolymers.

| Sample | Integration ratios | | | | Final molar composition PEG: PPG: PCL: Glycerol |
| --- | --- | --- | --- | --- | --- |
|  | PEG | PPG | PCL | Glycerol |  |
| EPC(3:1) G0.25 | 171 | 27.3 | 0.360 | 0.330 | 3.48: 1.00 : 0.0415: 0.315 |
| EPC(3:1) G0.5 | 190 | 32.8 | 0.400 | 0.600 | 3.55: 1.00: 0.0419: 0.518 |
| EPC(3:1) G0.75 | 180 | 28.4 | 0.370 | 0.850 | 3.52: 1.00: 0.0413: 0.768 |
| EPC(2:1) G0.25 | 230 | 55.6 | 0.640 | 0.360 | 2.30: 1.00: 0.0358 : 0.166 |
| EPC(2:1) G0.5 | 245 | 54.5 | 0.540 | 0.880 | 2.50: 1.00: 0.0308: 0.416 |

**Table S4**: Summary of critical micelle concentrations (CMC) of the EPCG copolymers.

| Sample | CMC at 15^o^C  (wt %) | CMC at 25^o^C  (wt %) | CMC at 45^o^C  (wt %) |
| --- | --- | --- | --- |
| EPC(3:1) G0.25 | 0.267 | 0.0784 | 0.0162 |
| EPC(3:1) G0.5 | 0.224 | 0.100 | 0.0183 |
| EPC(3:1) G0.75 | 0.166 | 0.106 | 0.0219 |
| EPC(2:1) G0.25 | 0.0877 | 0.0515 | 0.0104 |
| EPC(2:1) G0.5 | 0.279 | 0.0936 | 0.0317 |
| EPC(2:1) G0.75 | 0.0830 | 0.0516 | 0.0108 |

## Supplementary Figures


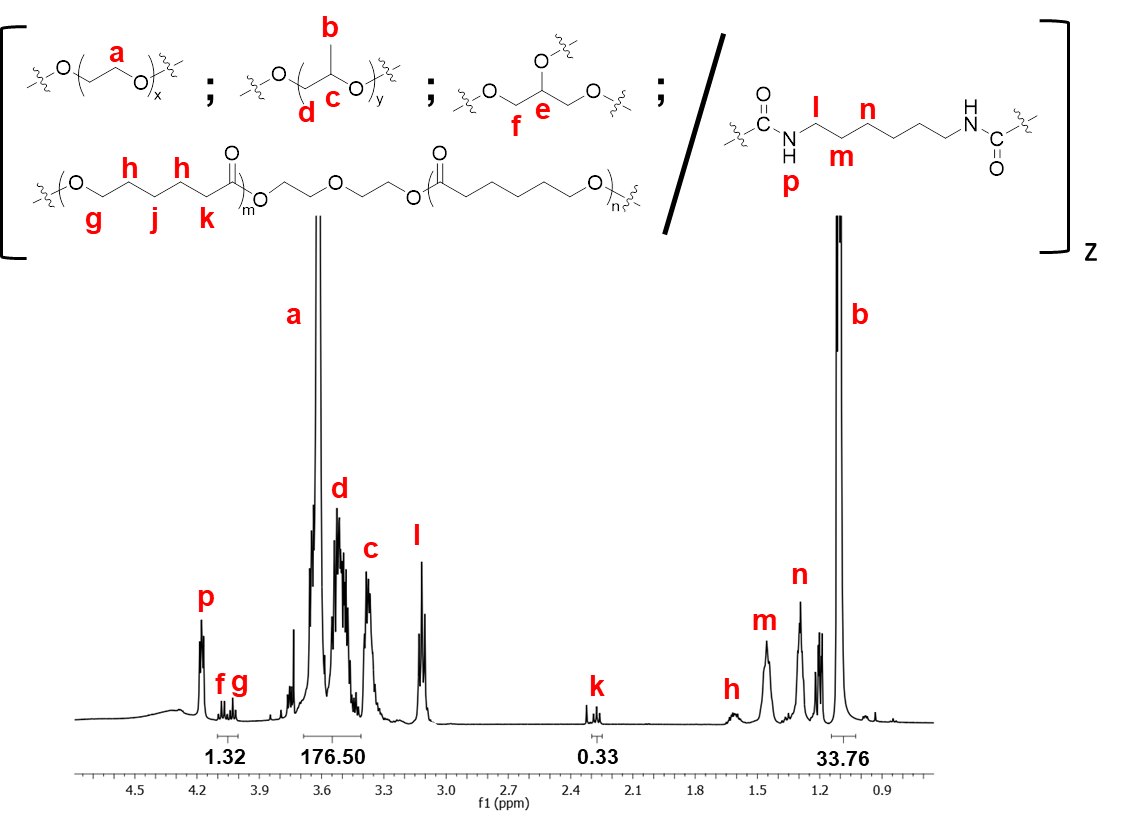


**Figure S1**: ^1^HNMR spectrum of representative EPCG copolymer (EPC(2:1) G0.75) in CDCl_3_ labelled with corresponding protons and integration ratios of characteristic peaks. Peak labelled “e” may be hidden under the large peaks seen in the range of 3.3 – 3.7 ppm.


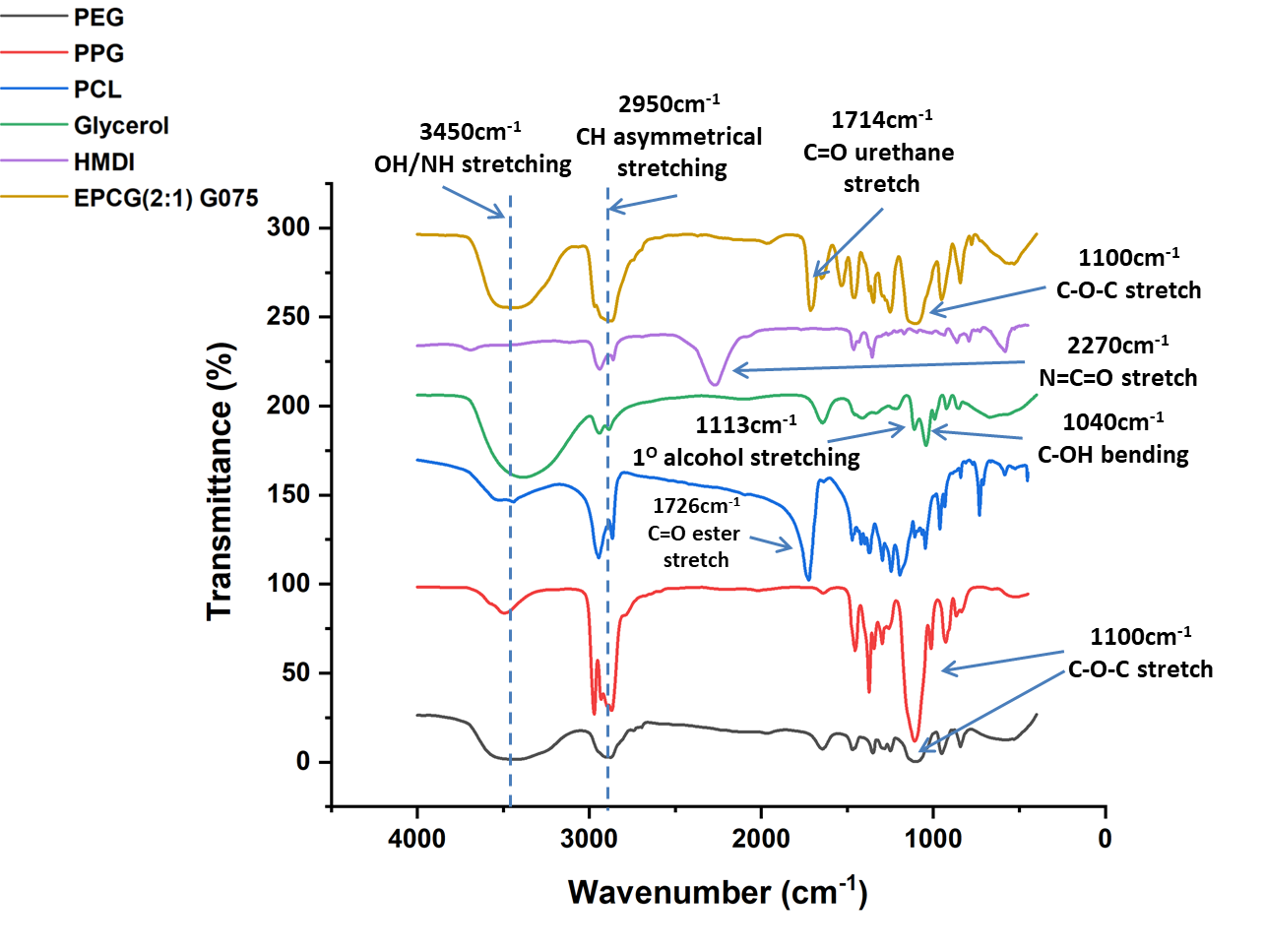


**Figure S2**: FTIR spectrum of representative EPCG copolymer


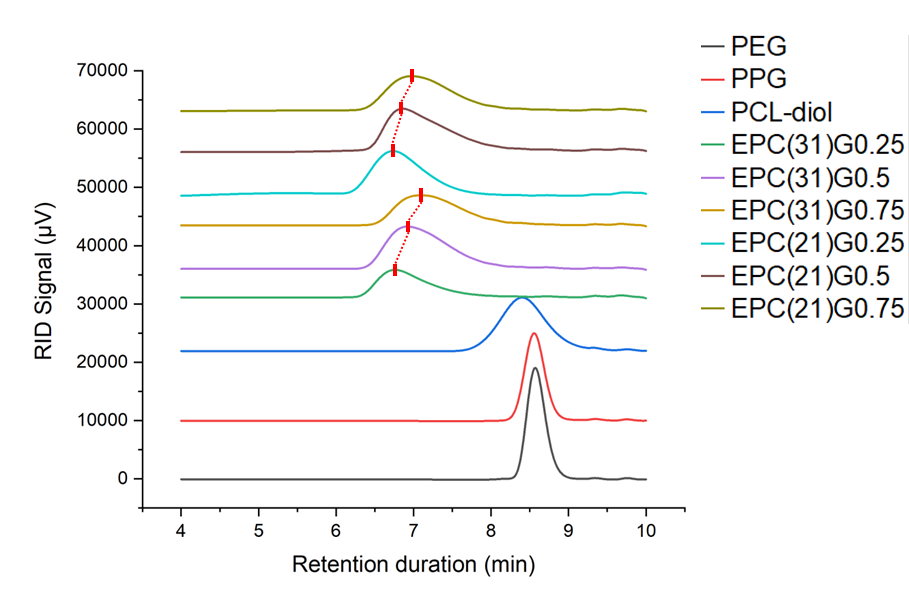


**Figure S3**: GPC spectrums of EPCG copolymers


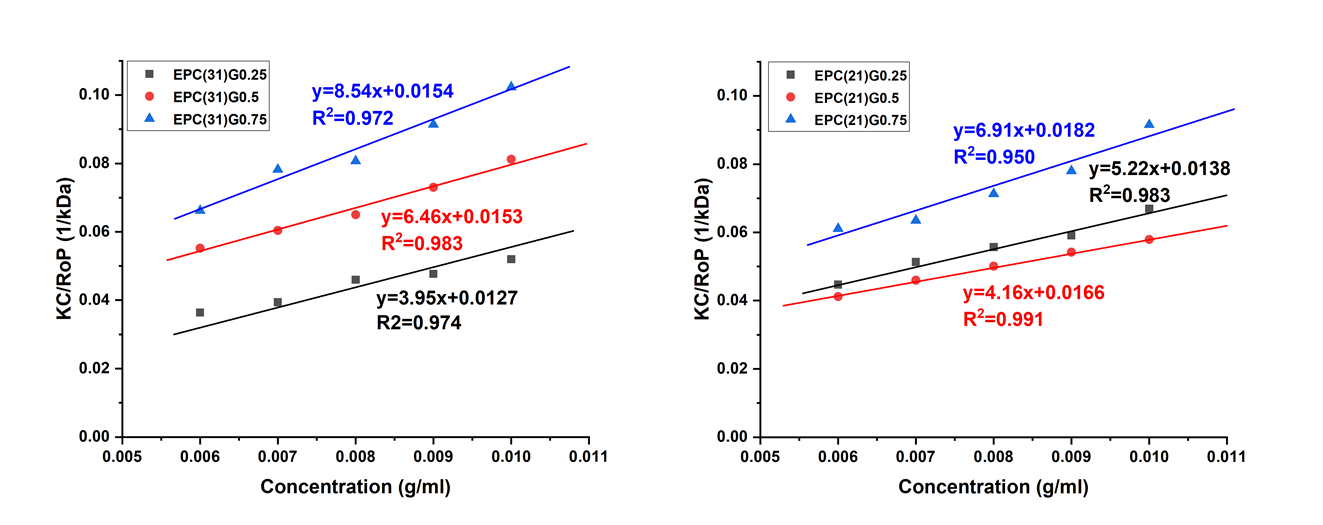


**Figure S4**: Debye plots of EPCG copolymers obtained from static light scattering (SLS).


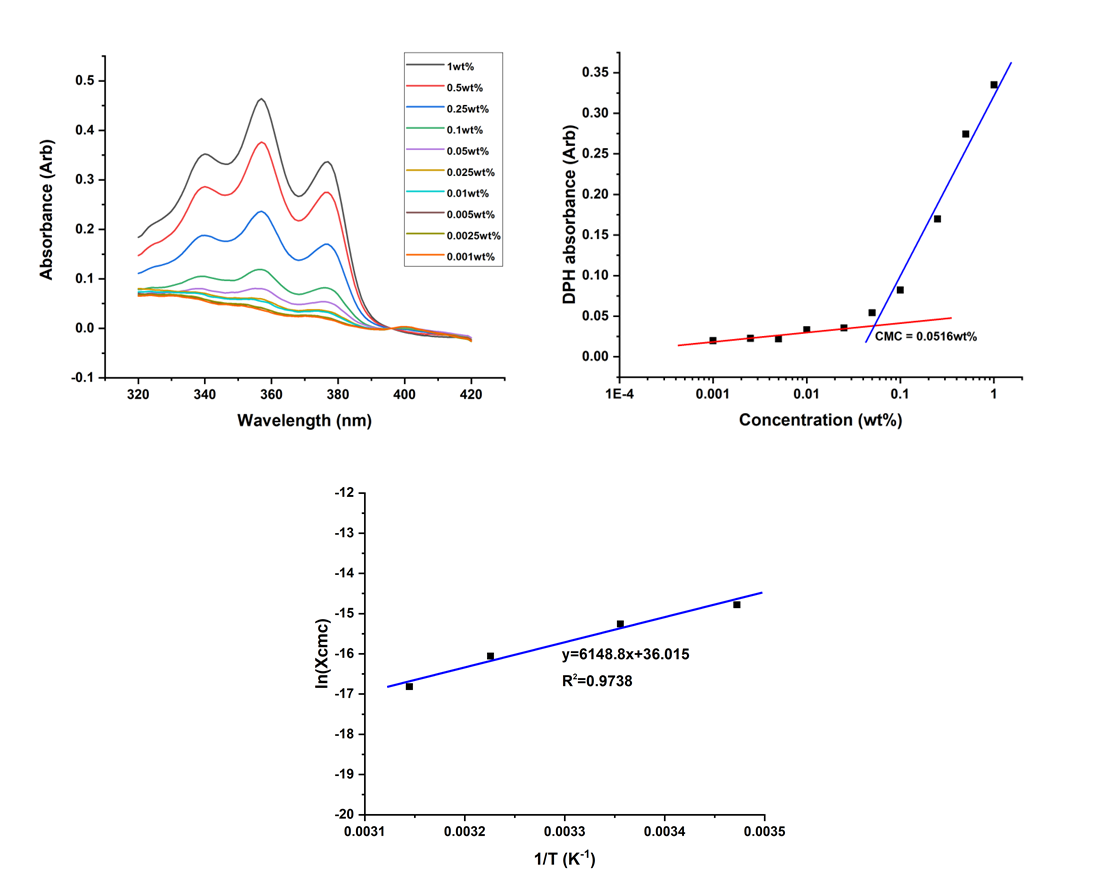


**Figure S5**: A) DPH absorbance in micellar solutions, B) CMC calculation, and C) Arrhenius plot for representative EPCG copolymer (EPC(2:1) G0.75)).

**
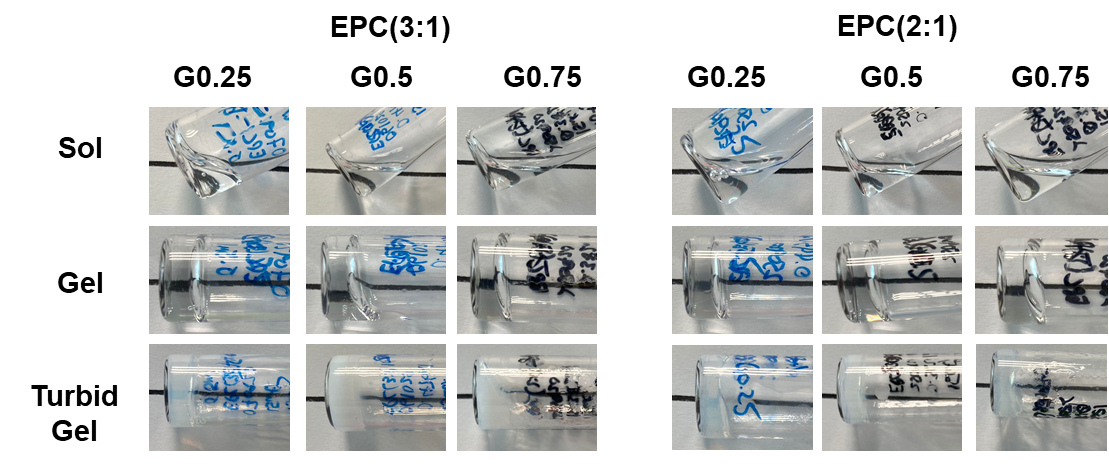
**

**Figure S6**: Photo panel of EPCG gels at 15wt% in the sol, gel, and turbid gel phases at 4^o^C, 37^o^C, and 70^o^C respectively. All EPCG gels formed were optically clear.


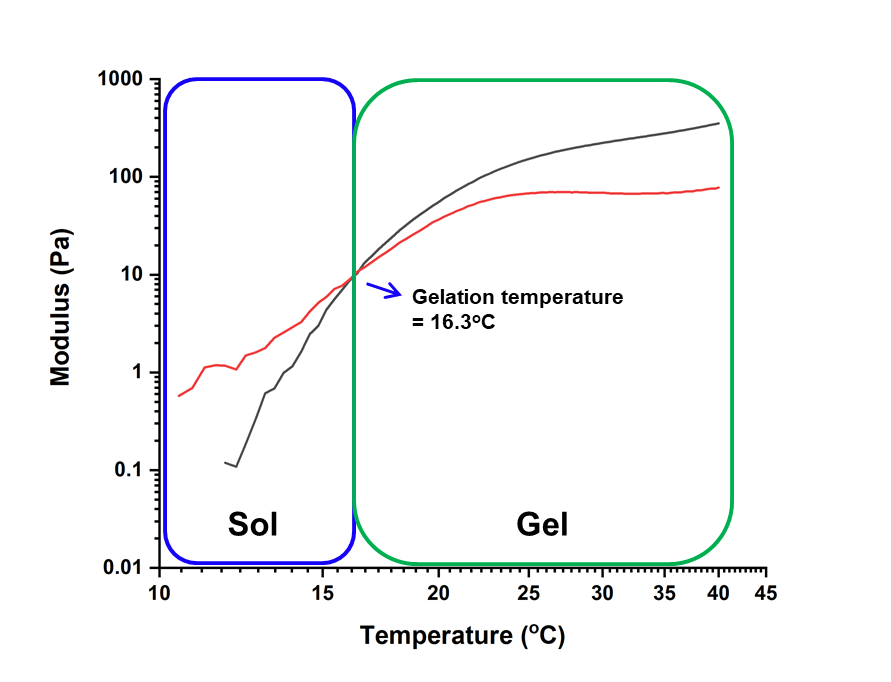


**G”**

**G’**

**Figure S7**: Oscillatory rheological temperature sweep of representative EPCG thermogel (EPC(2:1) G0.75) showing sol-gel phase transition.


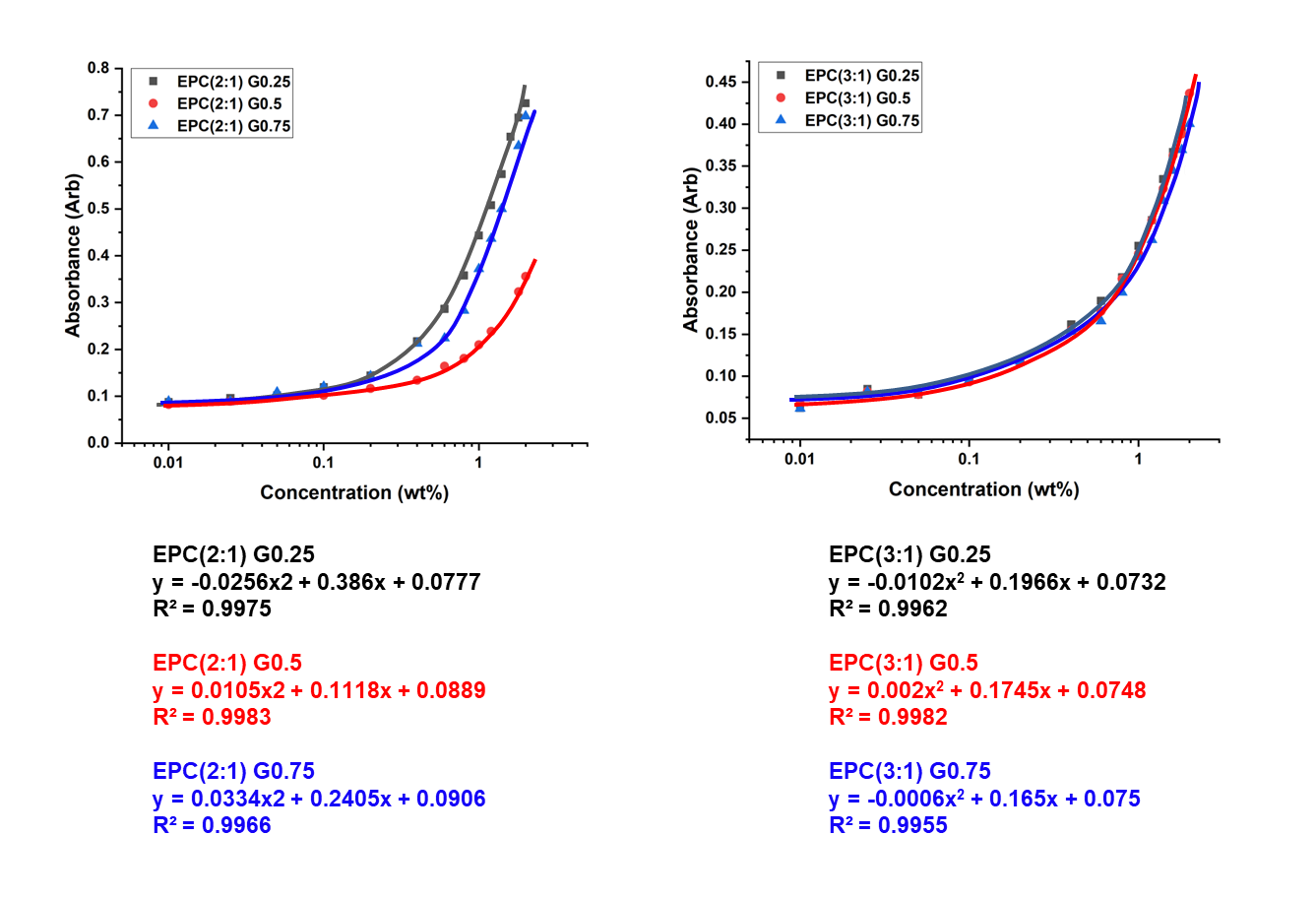


**Figure S8**: Standard curves relating DPH absorbance to micelle concentration.


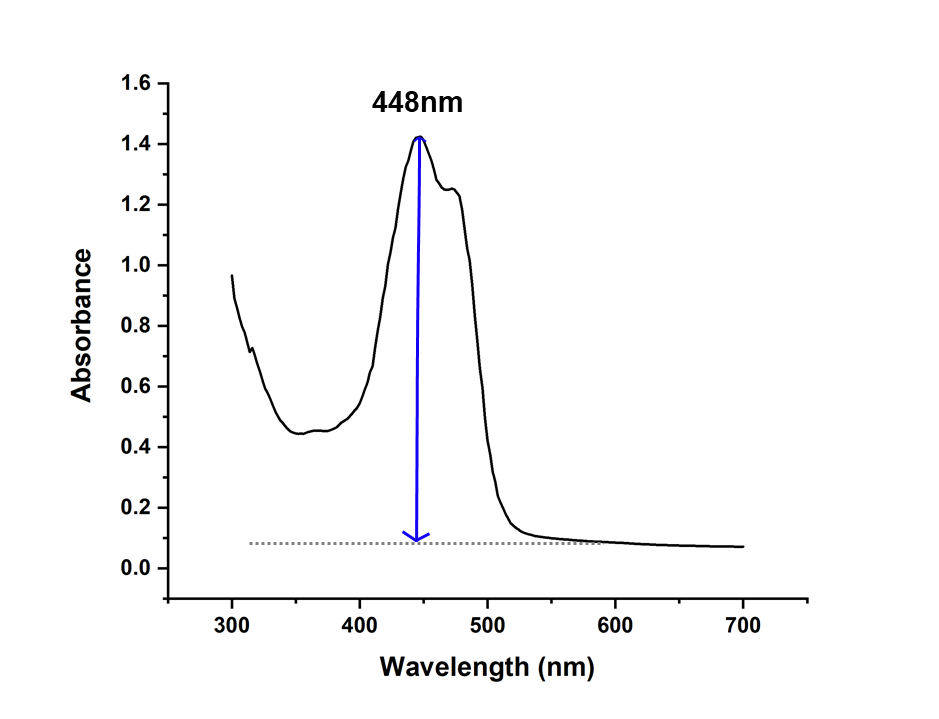


**Figure S9**: Absorbance spectrum of fluorescein in water.


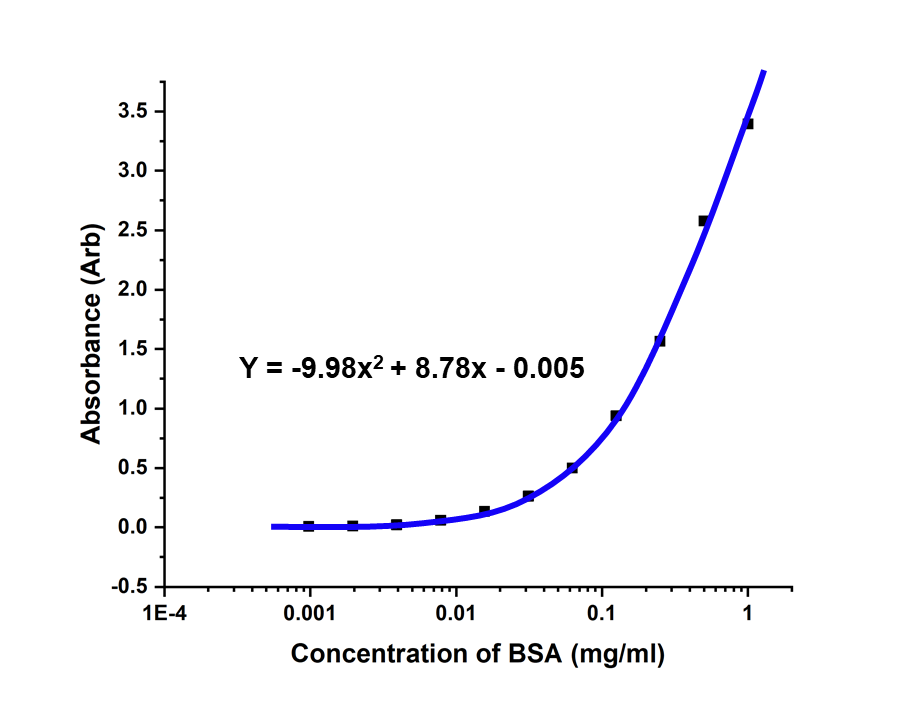


**Figure S10**: Standard curve relating absorbance to BSA concentration.
